# Dopamine alters phage morphology to exert an anti-infection effect

**DOI:** 10.3389/fmicb.2023.1272447

**Published:** 2023-11-09

**Authors:** Shengting Zhang, Xiuling Hu, Chunting Zhang, Yani Ju, Xin Liu, Yunlin Wei

**Affiliations:** ^1^School of Ethnic Medicine, Yunnan Minzu University, Kunming, China; ^2^Faculty of Life Science and Technology, Kunming University of Science and Technology, Kunming, China; ^3^Faculty of Narcotics Control, Yunnan Police College, Kunming, China

**Keywords:** anti-infection, anti-phage activity, bacteriophage, catecholamine, dopamine, quantitative RT-PCR

## Abstract

Antiviral drug development is important for human health, and the emergence of novel COVID-19 variants has seriously affected human lives and safety. A bacteriophage—a bacterial virus with a small and simple structure—is an ideal experimental candidate for studying the interactions between viruses and their hosts. In this study, the effects and mechanisms of catecholamines on phages were explored, and dopamine (DA) was found to have general and efficient anti-infection effects. A clear dose-dependent effect was observed when different phages were treated with DA, with higher DA concentrations exhibiting stronger anti-phage activity. The half maximal inhibitory concentration values of DA for vB-EcoS-IME167, T4 Phage, and VMY22 were determined as 0.26, 0.12, and 0.73 mg mL^−1^, respectively. The anti-phage effect of DA increased with treatment duration. In addition, the anti-infection activities of DA against vB-EcoS-IME167, T4 Phage, and VMY22 were increased by 10^5^, 10^4^, and 10^4^ folds compared to that of the control. This ability of DA was observed only in phages and not in the host bacteria. Morphological changes of phages were observed under transmission electron microscopy following their treatment with DA, and considerable changes in adsorption were confirmed *via* quantitative reverse transcription polymerase chain reaction. These results suggest that the anti-phage effect of DA is primarily due to the destruction of the external structure of the phage. This study, to the best of our knowledge, is the first to report the universal anti-phage infection effect of dopamine, which provides novel information regarding DA and forms a basis for further research and development of antiviral drugs. Moreover, it provides a new perspective for the research about the defense and counter-defense of bacteria and bacteriophages.

## 1. Introduction

Viruses infect bacteria, fungi, actinomycetes, and other microorganisms (Bettarel et al., 2004; Ackermann, 2007; De La Higuera and Lázaro, 2022) and represent some of the most abundant members of the biosphere (Davies et al., 2016). The phage performs its own proliferation and progeny reproduction by lysing the host; it is a bacterial virus that specifically infects the host bacteria. Bacteriophages are also the most abundant viruses and possess low toxicity, presenting an ideal alternative biological model for viral studies that can be used in preliminary research for novel antiviral drug development.

Phages can be divided into virulent phages, which lyse the host, and lysogenic phages, which coexist with the host (Clokie et al., 2011; Hampton et al., 2020). Phage infections occur through five steps: adsorption, injection, synthesis, assembly, and release (Manning and Kuehn, 2011; Fortier and Sekulovic, 2013; Reyes-Robles et al., 2018). As bacteria and bacteriophages typically coexist in the biosphere, bacteria have evolved various defense mechanisms, such as adsorption inhibition, injection blocking, abortive infection, and restriction repair systems (Jackson et al., 2017; Tzipilevich et al., 2017; Hille et al., 2018; Gordeeva et al., 2019) to combat phage infection. The bacterial synthesis of dopamine (DA) has recently been reported (Villageliú and Lyte, 2018; Liu and Zhu, 2020). Therefore, studies on the anti-phage mechanisms of DA are necessary to expand our understanding of DA and provide new insights into the defense mechanisms of microbial systems.

Catecholamines are active substances that primarily function as neurotransmitters and hormones (Szopa et al., 2001; Eisenhofer et al., 2004). Sriram et al. found that catecholamines play an important role in the physiological regulation of the cardiovascular, respiratory, metabolic, and immune systems in COVID-19 (Gubbi et al., 2020). DA is an important neurotransmitter that is widely found in animals and plants; it regulates important functions and is the first component of the catecholamine synthesis pathway (Jiang et al., 2006; Juárez Olguín et al., 2016). DA can enhance adaptability to various stimuli (Norcliffe-Kaufmann, 2022) and improve plant resistance to pathogens and environmental stress (Allen, 2003; Skirycz et al., 2005). DA is a catecholamine comprising catechol and amino group side chains. In nature, catecholamines are active substances that have various physiological and biochemical effects on animals and plants (Li P. et al., 2019; Kaufmann et al., 2020); however, their effects on microorganisms have not been reported. Therefore, investigations are required to fill the existing research gap on catecholamines and their functions in microorganisms.

Catecholamine substances, including dopamine, are often present in environments where bacteria and bacteriophages coexist. Previous studies have found that they played important roles in bacterial growth, biofilm formation, etc. However, there have been no reports on the specific effects of dopamine on bacteriophages. The dopamine produced by bacteria itself is limited under normal growth; however, it can produce high concentrations of dopamine under environmental stress, which include biological, physical, and chemical factors. Therefore, it is necessary to explore the defense and counter-defense mechanisms of dopamine against bacteria and bacteriophages (Lyte and Ernst, 1992; Sharaff and Freestone, 2011).

In this study, three catecholamines [DA, norepinephrine (NE), and epinephrine (E)] and three phages (vB-EcoS-IME167, T4 phage, and VMY22) were used to perform a series of anti-phage infection studies. All bacteriophages used in this study were virulent. The results showed, for the first time, that DA had a general and efficient effect on blocking phage infection of host bacteria; the highest anti-infection abilities of vB-EcoS-IME167, T4 Phage, and VMY22 were 10^5^, 10^4^, and 10^4^ times that of the control, respectively. The anti-infection effect of DA was found to target only phages and preliminarily confirmed the anti-infection mechanism: DA decreased the phage adsorption rate, leading to inhibition of the adsorption step during infection. The findings of this study highlight the role of DA and catecholamines in their specific action toward phages and significantly contribute to further studies on antiviral drug development. DA is a kind of hormone produced by the host under stress or stress, which plays an important role in increasing bacterial susceptibility (Fan and Pedersen, 2021). It is an effective method for improving bacterial infections from the perspective of bacteriophages. Our findings provide a new perspective; however, research in this area remains lacking. The study of DA against bacteriophage infection is worth exploring. These results broaden the understanding of catecholamines and lay a foundation for further research and development of antiviral drugs, providing new insights for the in-depth exploration of the defense mechanism of microbial systems. The findings of this study highlight the role of DA and catecholamines in their specific action toward phages and significantly contribute to further studies on antiviral drug development.

## 2. Materials and methods

### 2.1. Bacterial strains, reagents, and culture conditions

DA, NE, and E were purchased from the Shanghai Aladdin Biochemical Technology Co., Ltd. (Shanghai, China). The raw materials for Luria-Bertani (LB) medium, Minimal Medium (MM), and experimental consumables were obtained from Yunnan Chien Technology Company (Kunming, China). All chemicals used in this study were of analytical grade. Ultrapure water was used to prepare sterile aqueous solvent (Milli-Q system, 18.2 M Ω cm, 25°C).

*Escherichia coli* BW25113, *E. coli* ATCC 11303, and *Bacillus cereus* MYB41-22 were the three host bacteria used in the experiment. The phages vB-Ecos-IME167, T4 phage, and VMY22, belonging to *Siphoviridae, Myoviridae*, and *Podoviridae*, respectively, were selected for this study.

### 2.2. Activation of host bacteria

The preservation strains of the host bacteria, *E. coli* BW25113, *E. coli* ATCC 11303, and *B. cereus* MYB41-22, were removed from the ultra-low temperature refrigerator at −80°C. First, the host bacteria were placed on crushed ice to thaw naturally. Second, a small volume of the bacterial preservation solution was transferred to a solid plate medium *via* plate streaking for bacterial activation. The plates were then marked and placed in the LB medium for bacterial culture (MM medium can only sustain the basic life processes of bacteria). Optimum culture temperatures were selected for the three host bacteria, namely, 37°C for *E. coli* BW25113 and *E. coli* ATCC 11303 and 28°C for *B. cereus* MYB41-22. After transfer, all bacteria were cultured overnight.

### 2.3. Preparation of bacteriophage

A single colony was selected from the activated host bacterial plates using an inoculation ring, and the bacterial solutions were expanded in a conical flask until the OD_600_ reached 0.8. The preserved phages were then added to the respective bacterial solutions and inoculated at a density of 10%. After the solutions turned clear, a sterile 0.22 μm stream filtration membrane was used to obtain each phage stock solution, which was marked and stored at 4°C for subsequent use.

### 2.4. Anti-phage activity assay

#### 2.4.1. Treatment concentration

Sterile water was used as the control, and different concentration gradients (0.01, 0.1, 1, 5, and 10 mg/mL) of each sample solution were mixed with the respective phage dilution at a ratio of 1:2. After 5 min of incubation, the effect of each sample on phage infection was compared *via* the double-layer plate method. The experiment was performed in triplicate. Semi-inhibitory concentration (IC_50_) values indicate half of the amount of a drug or inhibitor that inhibits certain substances, such as enzymes, cell receptors, or microorganisms. The ratio of DA concentration to inhibition rate was used to calculate the IC_50_, which reflects the anti-infective ability of DA more directly.

#### 2.4.2. Time of treatment

Sterile water was used as the control, the sample concentrations were determined as described previously, and different treatment times (10, 30, 60, 120, and 180 min) were applied. The differences between groups were statistically analyzed using the double-layer plate method. The experiment was performed in triplicate.

#### 2.4.3. Treatment environment

An anti-phage infection experiment was performed on the culture media to test the effects of LB and MM. The experiment was performed in triplicate. Briefly, three strains of host bacteria and three different bacteriophages were cultured in two different media: LB and MM (dilutions: 10^−1^, 10^−2^, 10^−3^, 10^−4^, 10–^5^, 10^−6^, 10^−7^). DA solution at a concentration of 10 mg/mL and bacteriophages at different dilutions were incubated for 60 min at a 1:2 ratio (100 μL DA + 200 μL bacteriophage). After incubation, each mixture was transferred to a 5 mL Eppendorf (EP) tube containing 200 μL of the host bacteria and was incubated for 5 min at 26°C (with three replicates per dilution tested). The sterilized semi-solid medium was dissolved in a microwave oven and then cooled to 55–65°C. After incubation, the bacterial mixture was quickly poured onto the solid medium and then cultured overnight at 37°C/ 28°C. The plaque on the plate was counted, and the pfu/mL was calculated.

### 2.5. Morphological properties based on transmission electron microscope (TEM) imaging

#### 2.5.1. Phage purification

The phage stock solution (500 mL) was expanded to obtain highly concentrated purified phage particles by enzymatic hydrolysis, centrifugation, filtration, sedimentation, and separation, as described below.

The plated host bacteria were inoculated in a 5 mL test tube and cultured overnight (with a 3% inoculation amount) in 500 mL LB liquid medium until OD_600_ = 1.0 was reached. Next, the host bacteria and corresponding phage were mixed at a ratio of 1:10 and cultured on a shaker at 180 rpm and 28°C/ 37°C until the solution turned clear. After cooling this solution to 20–25°C, 50 μL DNaseI and 50 μL RNase were added to digest the nucleic acids of host bacteria after cleavage. An amount of 29.2 g NaCl granules was dissolved into each sample bottle (placed in an ice bath) *via* stirring with a glass rod for 60 min. Next, the mixture was centrifuged (15 min, 4°C, 11000 × g) using a large cryo centrifuge. The supernatant was collected, its volume measured and placed in a 500 mL beaker. Polyethylene glycol (PEG_6000;_ 50 g/500 mL) was added according to the volume of the supernatant and dissolved with a magnetic agitator, and the mixture was allowed to settle overnight in an ice bath. Thereafter, the mixture was centrifuged again (15 min, 4°C, 11000 × g), and the supernatant was discarded. The phage precipitate was retained and resuspended in an SM solution (5.8 g/L NaCl, 2 g/L MgSO_4_·7H_2_O, 50 mL/L 1 mol/L Tris-Cl) at a proportion of 500 mL precipitate per 8 mL of SM solution. This suspension was incubated for 60 min at room temperature, and the resuspension liquid was transferred to a 50 mL centrifuge tube. An equal volume of chloroform was added. The remaining PEG_6000_ and host cell fragments were extracted, mixed evenly, and centrifuged at 3000 × g for 15 min. Its organic and hydrophilic phases were separated, retaining the hydrophilic phase containing phage particles. After measuring the volume thereof, cesium chloride was added at a proportion of 0.75 g/mL and gently shaken until dissolved. The mixture was centrifuged at 4°C and 160,000 × *g* for 12 h. Lastly, the ultra-free tube was carefully removed, and the concentrated phage particles were extracted with a syringe and stored at 4°C.

#### 2.5.2. Negative dyeing

Using a pipette gun, 20 μL of phage particles was gently aspirated into the same volume of different concentrations of DA solution (0, 0.01, and 10 mg/mL). After incubation for 60 min, 5 μL of the mixture was absorbed and gently added to the carbon-coated layer of a copper mesh, which was then kept static to allow adsorption for 10 min at room temperature under gravity. The remaining liquid on the copper mesh was blotted with filter paper and allowed to dry for 1 min, after which 5 μL of 1% phosphotungstic acid solution was added for 2 min.

#### 2.5.3. TEM imaging

TEM images were used to observe changes in the morphology of phages treated with DA. A purified and high concentration of each bacteriophage (20–100 μL) was gently poured into a 1.5 mL EP tube with a liquid transfer gun. It was incubated with 20 μL of ddH_2_O and 0.01 or 10 mg/ mL of DA solution for 60 min. Next, 5 μL of the mixture was gently placed on a carbon-coated copper mesh grid and allowed to adsorb statically through self-gravity for 10 min at room temperature. Thereafter, any remaining liquid was removed from the copper mesh with clean filter paper, and the sample was re-dyed with 5 μL of 1% phosphotungstic acid solution for 2 min. The remaining liquid was once again absorbed with a clean filter paper, and the samples were observed under a projection electron microscope (Tecnai G2 TF30 S-Twin; FEI, Hillsboro, OR, United States).

### 2.6. Quantitative reverse transcription polymerase chain reaction (RT-qPCR)

The whole phage genome was extracted using a Viral DNA Kit (Omega Biotek, GA, United States), a recombinant plasmid was prepared, and a standard curve was constructed. Using sterile water as the control and DA as the experimental group, the phage solution was mixed at a ratio of 9:1, incubated for 30 min, centrifuged, and filtered, with the supernatant being retained. The phage genome was extracted from the supernatant and amplified *via* RT-PCR. The results of the experimental and control groups were compared based on the standard curve. The Ct value was used as the horizontal coordinate, and the logarithm of the standard copy number was used as the vertical coordinate to create the standard curve (Fig. S1). Three genes were selected: the tail fiber protein of vB-EcoS-IME167, a short-tail fiber protein of the T4 phage, and DNA packaging ATPase in VMY22.

The vB-EcoS-IME167 phage was represented as $Y=-0.2968x+12.94,R^{2}=0.9991\text{.}$

The T4 phage was represented as $Y=-0.3754x+10.218,R^{2}=0.9991$

VMY22 was represented as $Y=-0.3216x+7.7848,R^{2}=0.9994$

### 2.7. Statistical analysis

Data represent the mean ± standard error (SE) of at least three experiments done in triplicate (n = 3). GraphPad Prism 9.5.1 software was used for all analyses. Paired t-tests were used to compare data between treated and untreated groups and to compare means within the same set of experiments. Results were considered statistically significant at *p* < 0.05 (*p < 0.05, **p < 0.01, ***p < 0.001, ****p < 0.0001).

## 3. Results

### 3.1. Anti-phage experiment

**Figure 1:**
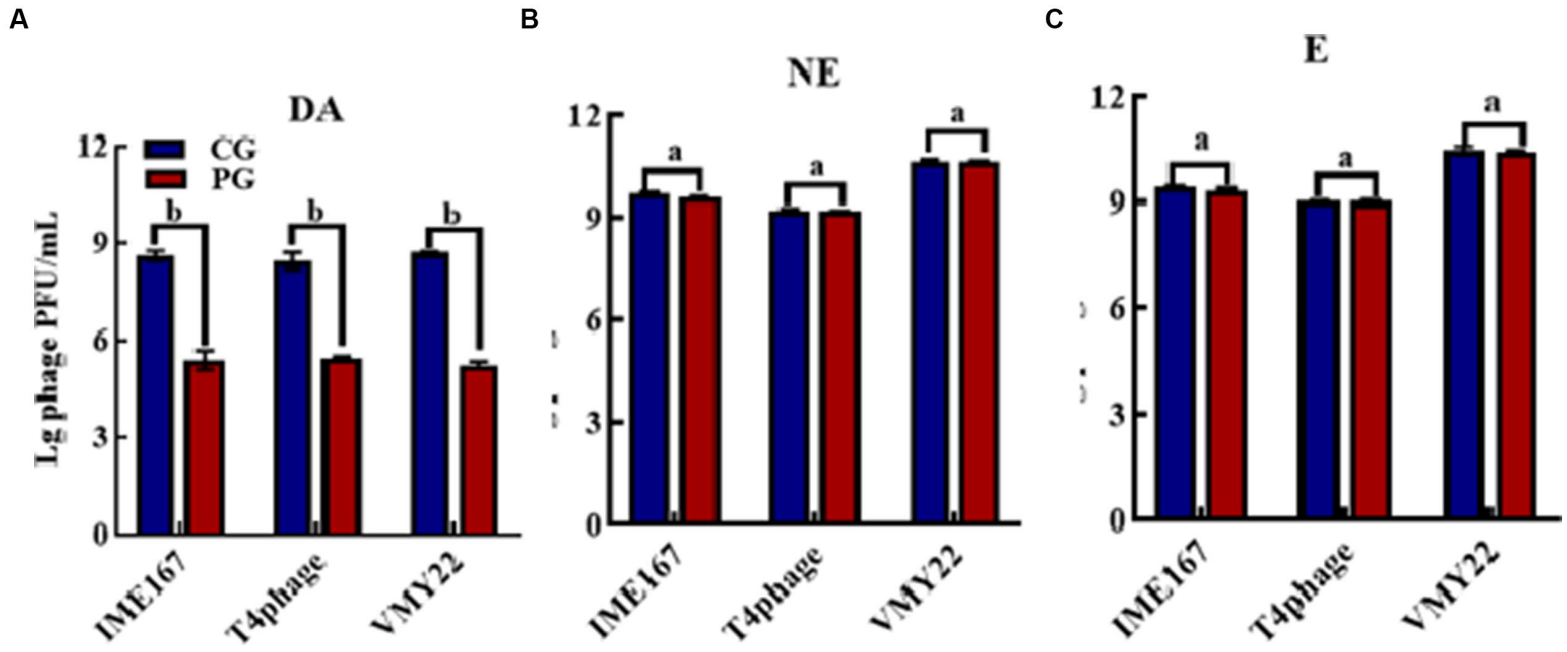
Anti-phage activities of different reagents. **(A–C)** indicate resistance to dopamine (DA), norepinephrine (NE), and epinephrine (E). (CG, control group; PG, treatment group). Error bars indicate the standard deviation of three replicate cultures. Different letters indicate significant differences (one-way Prism with Tukey’s *post hoc* test; a: *p* > 0.05, *p* < 0.05).

The anti-phage activities of DA, NE, and E are shown in Figure 1. DA showed a significant anti-phage effect, whereas NE and E had no effect on phage activity compared with the control. Indeed, DA showed a more than 10^3^-fold inhibitory effect on all three phages tested. Thus, DA exhibited a high inhibitory activity against phage infection.

### 3.2. Variation in inhibition based on concentration

**Figure 2:**
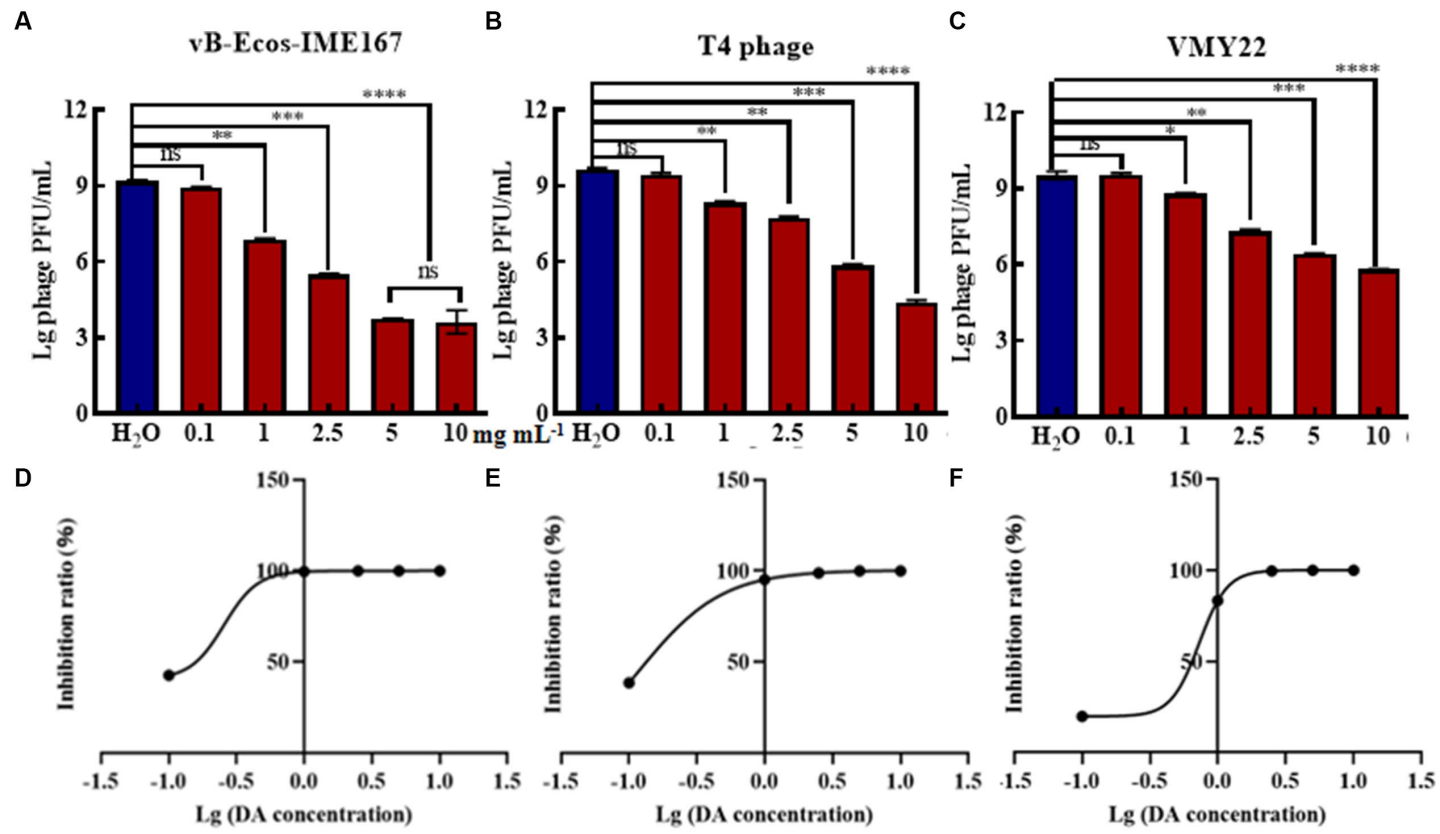
The different concentrations of DA and the IC_50_ of DA. **(A–C)** show three different phages, vB-EcoS-IME167, T4, and VMY22, treated with different concentrations of DA. Error bars indicate the standard deviation of three replicate cultures. Different letters indicate significant differences (one-way ANOVA with Tukey’s *post hoc* test; *p < 0.05, **p < 0.01, ***p < 0.001, and ****p < 0.0001). **(D–F)** indicate the half-maximal inhibitory concentrations (IC_50_) of DA against vB-EcoS-IME167, T4 phage, and VMY22; the IC_50_ value was calculated using the ratio of the logarithm of the DA concentration to the inhibition rate.

Due to the high efficiency of DA against phage infection in host bacteria, we performed gradient experiments to determine the optimal treatment conditions. The three phages exhibited a dose-dependent relationship, and their anti-infection abilities increased with increasing phage concentration. The strongest resistance to vB-EcoS-IME167 was observed at 5 mg/mL DA, and the strongest resistance to the T4 phage and VMY22 was observed at 10 mg/mL DA. Further data mining of DA concentration and anti-infection ability is shown in Figures 2D–F. The semi-inhibitory concentrations (IC_50_) of the three phages were 0.26, 0.12, and 0.73 mg/mL, respectively. DA concentration was found to be the key factor affecting anti-infection ability. At a concentration of 10 mg/mL, the maximum resistance to vB-EcoS-IME167, the T4 phage, and VMY22 were 10^5^-, 10^5^-, and 10^4^-fold, respectively, and the lowest effective concentration of DA was in the range of 0.1–1 mg/mL, revealing the efficient and extensive ability of DA to inhibit phage infection in bacteria.

### 3.3. Variation in inhibition based on treatment duration

**Figure 3:**
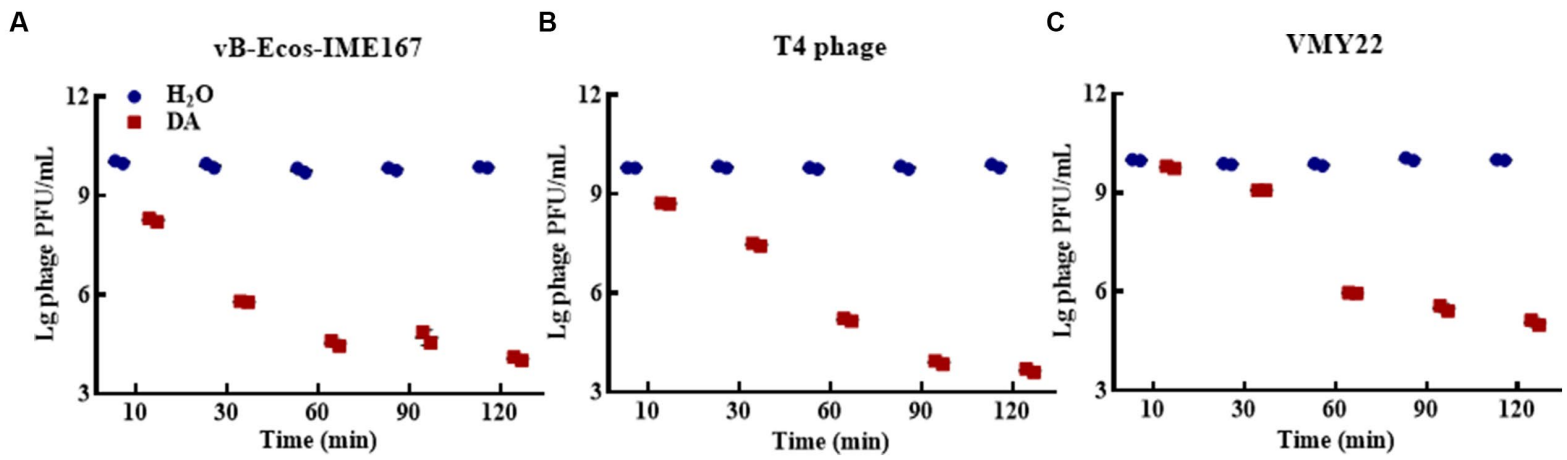
The different incubation times of DA. **(A–C)** show anti-phage activity against vB-EcoS-IME167, T4 phage, and VMY22, respectively. Bacteriophages vB-EcoS-IME167, T4 phage, and VMY22 were treated with DA for 10, 30, 60, 90, or 120 min. The plaque number was recorded using a double-layered plate and plaque forming units (pfu/mL) were calculated using the formula (pfu/mL = plaque number × 10 × dilution times); the experiment was performed in triplicate.

Different DA treatment times induced diverse anti-phage activity intensities. The experimental results for DA-induced resistance to phages at incubation times of 10, 3-, 60, 90, and 120 min are shown in Figure 3. Compared to that of the control, the anti-phage activity of DA was enhanced with longer treatments. At 10 min, DA showed an approximately 10-fold ability to block vB-EcoS-IME167 and T4 phage infection in host bacteria. For VMY22, DA instantaneously exhibited a 10^3^-fold resistance at 60 min. From the overall trend depicted in Figure 3, it is evident that DA exhibited rapid anti-infection activity against vB-EcoS-IME167 and T4 phages within a short period; this anti-infection ability increased with time, finally reaching approximately 10^4^-fold resistance. Simultaneously, DA-induced resistance in VMY22 rapidly increased by more than 10^3^-fold between 30 and 60 min and then stabilized. The duration of DA treatment is therefore a key factor in resistance. The maximum inhibitory activity of a variety of phages also corresponded with varied treatment times.

### 3.4. Inhibition effect of environments

**Figure 4:**
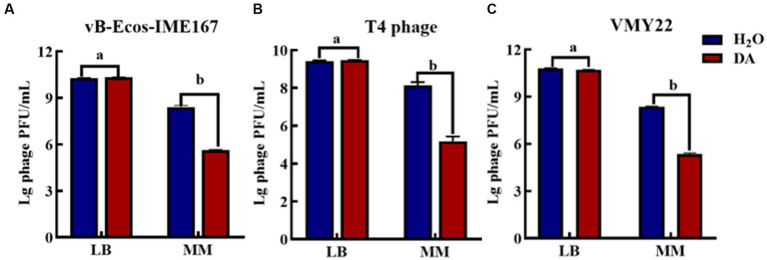
The anti-phage effect of DA treatment in different media. **(A–C)** show the anti-phage activity when phages were treated with DA in Luria-Bertani (LB) or minimal (MM) media. Bacteriophage concentrations were diluted in LB and MM media. After receiving DA treatment, these bacteriophages were introduced to a bacterial solution. The plaque number was recorded using the double-layer plate method and the plaque forming units (pfu) were calculated using the following formula: pfu/mL = plaque number × 10 × dilution times. Error bars indicate the standard deviations of three replicate cultures. Different letters indicate significant differences (one-way Prism with Tukey’s *post hoc* test; a: *p* > 0.05, b: *p* < 0.05).

Microbial growth generally places strict requirements on culture media. Therefore, we selected LB and MM, which are commonly used for microbial growth, to confirm the influence of the cultivation environment on phage infection. Figure 4 shows that the three phages responded the same after receiving either DA or H2O (control) treatment in LB, indicating that the anti-infection effect of DA was negligible in this environment. However, DA induced a 10^3^-, 10^3^-, and 10^2^-fold higher resistance to the three respective phages in an MM environment than the control treatment, indicating that the DA treatment of phages in MM led to a significant decline in phage infection activity. We speculate that the complex composition of LB media and the complex macromolecules contained in yeast powder and peptone may affect the action of DA on phages, thus resulting in this phenomenon; however, the composition of MM is simpler, mostly comprising inorganic ions and has relatively little influence on the interaction between DA and phages. Therefore, the MM medium may allow the effective inhibition of phages *via* DA.

### 3.5. The illustration of mechanisms involved in phage resistance of DA

**Figure 5:**
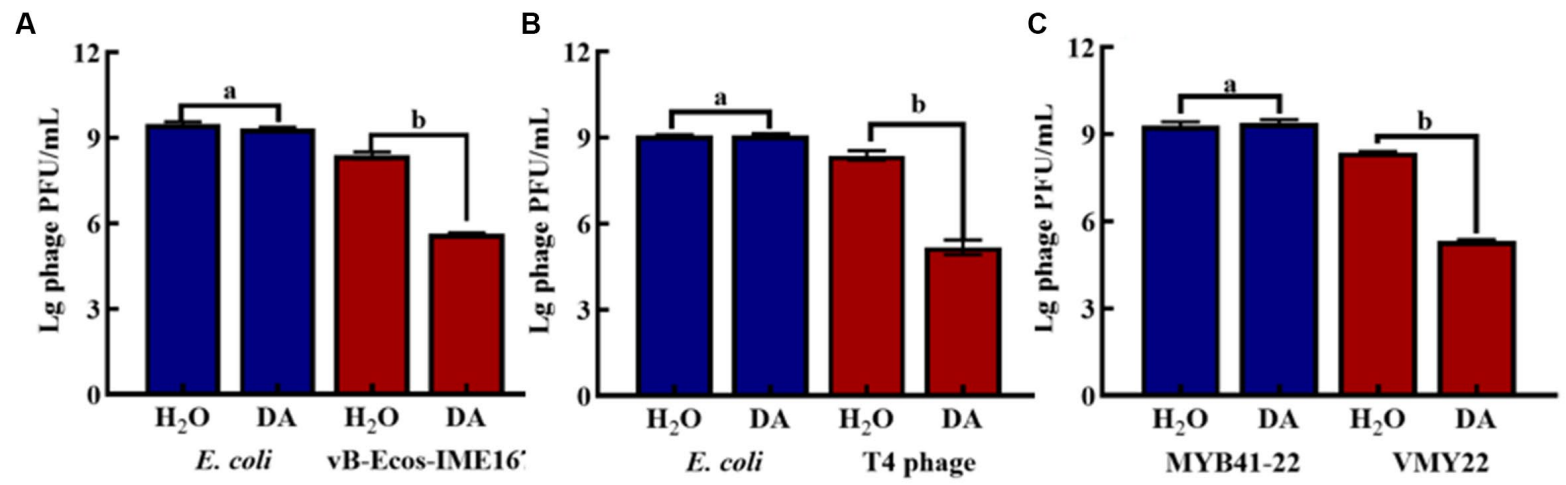
The effect of anti-phage activity of DA under different cultivation conditions. **(A–C)** show results for host bacteria and phages that were treated with either DA or sterile water (control). Phage spots were recorded using the double-layer plate method, and plaque forming units (pfu) were calculated using the following formula: pfu/mL = plaque number × 10 × dilution times. Error bars indicate the standard deviations of three replicate cultures. Different letters indicate significant differences (one-way Prism with Tukey’s *post hoc* test; a: *p* > 0.05, b: *p* < 0.05).

DA was found to induce resistance to bacteriophage infections only in the MM environment. The main target of DA remained unclear and could have been the host bacteria or bacteriophages. Hence, we compared the plaque-forming units of host bacteria and bacteriophages treated with either DA or sterile water (control) in MM (Figure 5). No significant difference was observed in the number of plaques between the experimental (DA) and control (sterile water) groups of host bacteria. In contrast, the experimental group of bacteriophages showed a considerable 10^2^-fold decrease in plaque-forming units following DA treatment compared to the control. These results show that the anti-infective effect of DA is seen only during its action on the phage and has no effect on the host bacteria, which further indicates that DA may cause some changes in the phage, leading to a sudden decline in its infection ability.

### 3.6. TEM imaging

**Figure 6:**
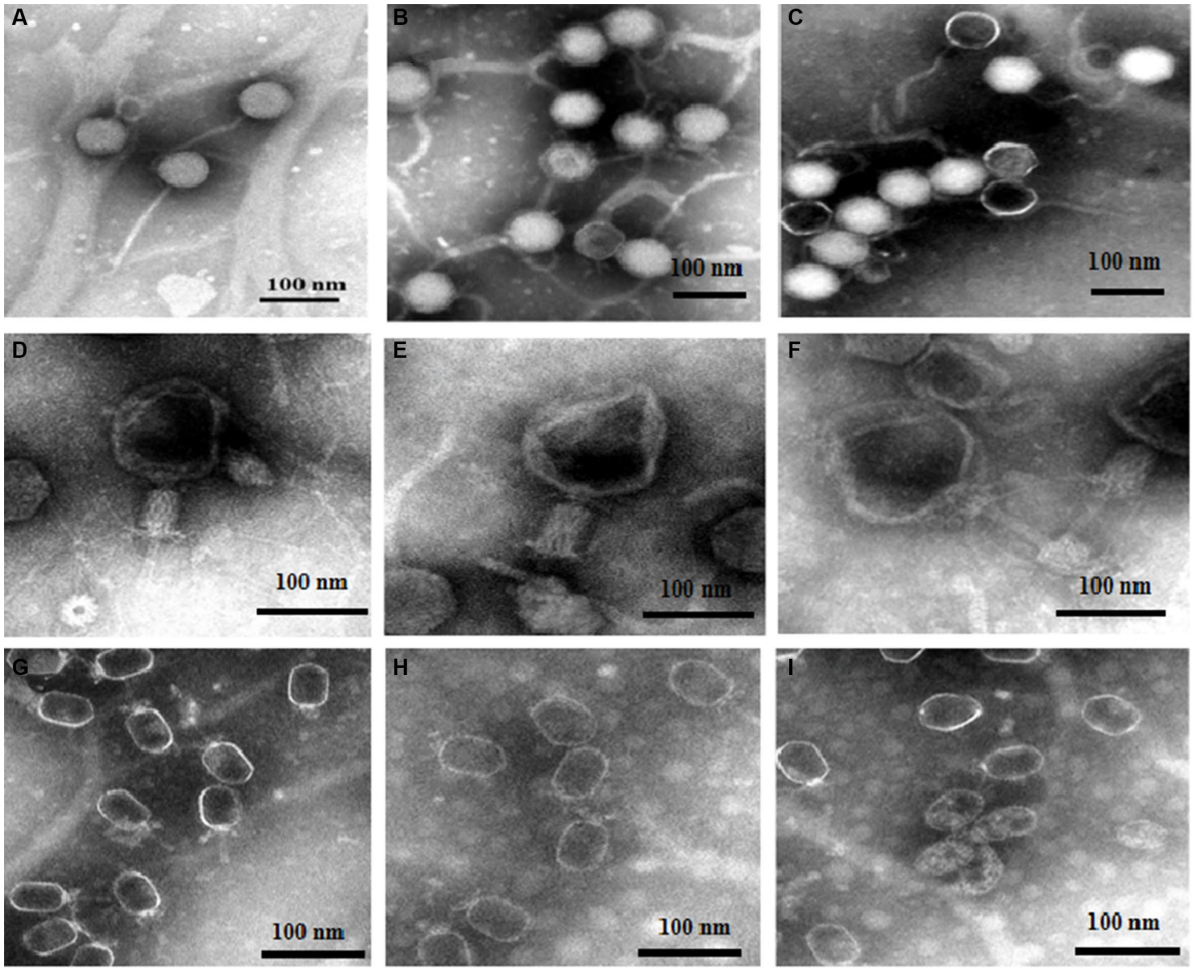
The morphological properties of different phages treated with DA under TEM. Scanning of the morphology and appearance of the three bacteriophages in different treatment groups was performed using transmission electron microscopy. **(A–C)** TEM images of vB-EcoS-IME167; **(D–F)** TEM images of the T4 phage; and **(G–I)** TEM images of VMY22. **(A)**, **(D)**, and **(G)** TEM images of phages in sterile water, **(B)**, **(E)**, and **(H)** at low concentrations of DA, and **(C)**, **(F)**, and **(I)** at high concentrations of DA.

Based on the above experiments, we speculated that DA caused changes in phages that resulted in a decrease in the adsorption rate. The morphologies of the three phages (treated with either DA or a sterile water control) were detected using TEM. Figures 6A–C show the resultant images of the structure of vB-EcoS-IME167 treated with sterile water, low DA concentrations, and high DA concentrations, respectively. Figure 6A (sterile water treatment) shows a slender phage with good integrity, with a head measuring approximately 40 × 40 nm and a slender tail approximately 150 nm in length. These characteristics are consistent with those reported in previous studies (Li Y. et al., 2019). Figure 6B (low DA exposure) shows an evidently blurred head and a cross-linked tail of the phage. In Figure 6C (high DA exposure), the phage displays an aggregated structure in which the head is connected, and the tail is tightly wrapped around it. Overall, DA significantly altered the morphological characteristics of vB-EcoS-IME167.

TEM images of the T4 phage exposed to similar treatments (Figures 6D–F) showed that, although the phages were fixed in the contraction state of the caudal sheath, they still had complete components of the T4 phage following treatment with sterile water (Figure 6D), including the caudal sheath, caudal tube, substrate, caudal tail, and caudal filaments of the head, neck, and tail (Ye et al., 2019). At low DA concentrations (Figure 6E), the head of the phage showed evident deformation, and the tail filaments and original complete morphology were lost. At high DA concentrations (Figure 6F), an image of a completely deformed T4 phase was obtained. The head was completely deformed and broken to release inclusions, the head and tail began to separate, most of the tail protein was deformed and fell off, and the phage decomposed. The morphological characteristics of the T4 phage were significantly altered by DA, similar to the results observed for vB-EcoS-IME167.

Lastly, TEM images for VMY22 are presented in Figures 6G–I. Figure 6G (sterile water treatment) shows the distinguishable hexagonal prism-like head, collar-like neck, and short straight tail of VMY22. The head size is 60 × 35 nm, the neck collar protein size is approximately 10 nm, and the tail length is approximately 40 nm (Fang et al., 2013). As shown in Figure 6H, following low DA exposure, the short, straight tail of most VMY22 phages was missing, the head and neck proteins remained intact, and the morphology was incomplete. Figure 6I shows that, after exposure to high DA concentrations, only the deformed head shell of the VMY22 remained. Even the deformed head shell was gathered, shrunk, and broken, and no original morphological characteristics of the VMY22 phage were evident. These three images revealed that DA significantly affected the morphological characteristics of VMY22 phages.

TEM images of vB-EcoS-IME167, the T4 phage, and VMY22 showed that the phages were not resistant to DA. We speculate that the anti-phage activity of DA first affects the complete morphology of the phages and then obstructs the specific adsorption step during phage infection.

### 3.7. Quantitative RT-PCR analysis

**Figure 7:**
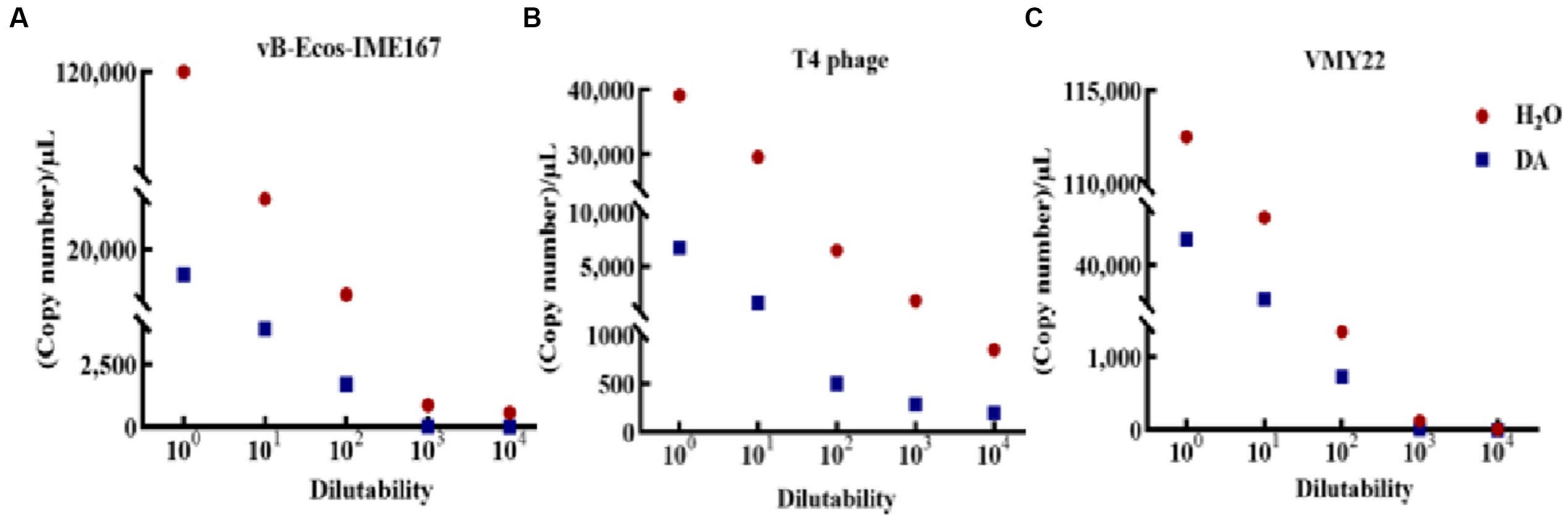
RT-PCR detection of the change of phage adsorption rate under DA treatment. The Ct values of the different treatment groups (sterile water: control group; DA: experimental group) were obtained using reverse transcription polymerase chain reaction (RT-PCR), and the corresponding copy numbers were obtained by substituting the standard curves of different phage recombinant plasmids (not shown). A lower copy number indicated a lower adsorption rate. **(A–C)** Changes in vB-EcoS-IME167, T4 phage, and VMY22 cells treated with DA and sterile water.

The accuracy of the hypothesized DA mechanism was further verified by comparing the changes in the adsorption rate using RT-PCR. As shown in Figure 7A, a significant difference was observed in the adsorption rate between DA-treated samples and the control. The copy number in DA-treated samples was lower than that in sterile water controls at each dilution, initially confirming that the anti-infection mechanism of DA on vB-EcoS-IME167 was caused by the destruction of specific adsorption. T4 phage and VMY22 also exhibited a similar phenomenon, with much lower DA than that of sterile water, indicating that DA could inhibit the specific adsorption of T4 phages and VMY22. These trends were consistent. Due to the resistance of DA to these three phages, the anti-phage activity of DA shows potential for application in the fermentation industry to control phage contamination.

## 4. Discussion

DA is an essential neurotransmitter in humans and a hormone-like reagent in plants and fungi. In this study, a potential anti-phage function of DA was discovered, and its mechanism was illustrated. This is the first report of the significant anti-phage activity of DA. The anti-phage activity of DA was found to be strictly dependent on its treatment time and dosage, and the lowest effective concentration of DA was 0.1–1 mg/mL. The half-maximal inhibitory concentrations of DA against phages vB-EcoS-IME167, T4, and VMY22 were 0.26, 0.12, and 0.73 mg mL^−1^, respectively. The anti-infection ability of DA was more than 10^4^-fold, and the resistance was significantly higher than that of previously reported antiviral agents (Ferro et al., 2018; Liao et al., 2020).

Currently, research on the relationship between catecholamines and microorganisms is primarily based on an increase in bacterial susceptibility to catecholamines (Halang et al., 2015); however, the anti-phage activity of DA has not been previously reported. In this study, we demonstrate, for the first time, that DA can significantly block phage infection in host bacteria. The TEM and RT-PCR results revealed that DA primarily affected bacteriophage morphology, thus reducing their adsorption rate onto bacteria. Most antiviral drugs achieve anti-infective effects by specifically acting on the adsorption process of the virus to host cells (Astani et al., 2012, 2014; Denzler et al., 2016). Approximately 50–70% of human DA originates from the digestive tract and is produced in large quantities by the intestinal flora. Studies have shown high concentrations of DA in the intestinal cavity: 10^−7^ to 10^−5^ mol/L in the pancreas, 10^−9^ to 10^−5^ mol/L in the small intestine, and 10^−8^ to 10^−4^ mol/L in the colon (Asano et al., 2012). In this study, a DA concentration of approximately 10^−4^ to 10^−3^ mol/L effectively blocked phage infection in bacterial hosts, which is slightly higher than the existing DA concentration in the intestinal environment; however, under stress, the local concentration may be much higher (Bansal et al., 2007). Upon infection, the local concentration of pathogens may be several orders of magnitude higher than the normal concentration, consistent with the effective concentration determined in this study (Lyte and Ernst, 1992; Lyte, 2004).

**Figure 8:**
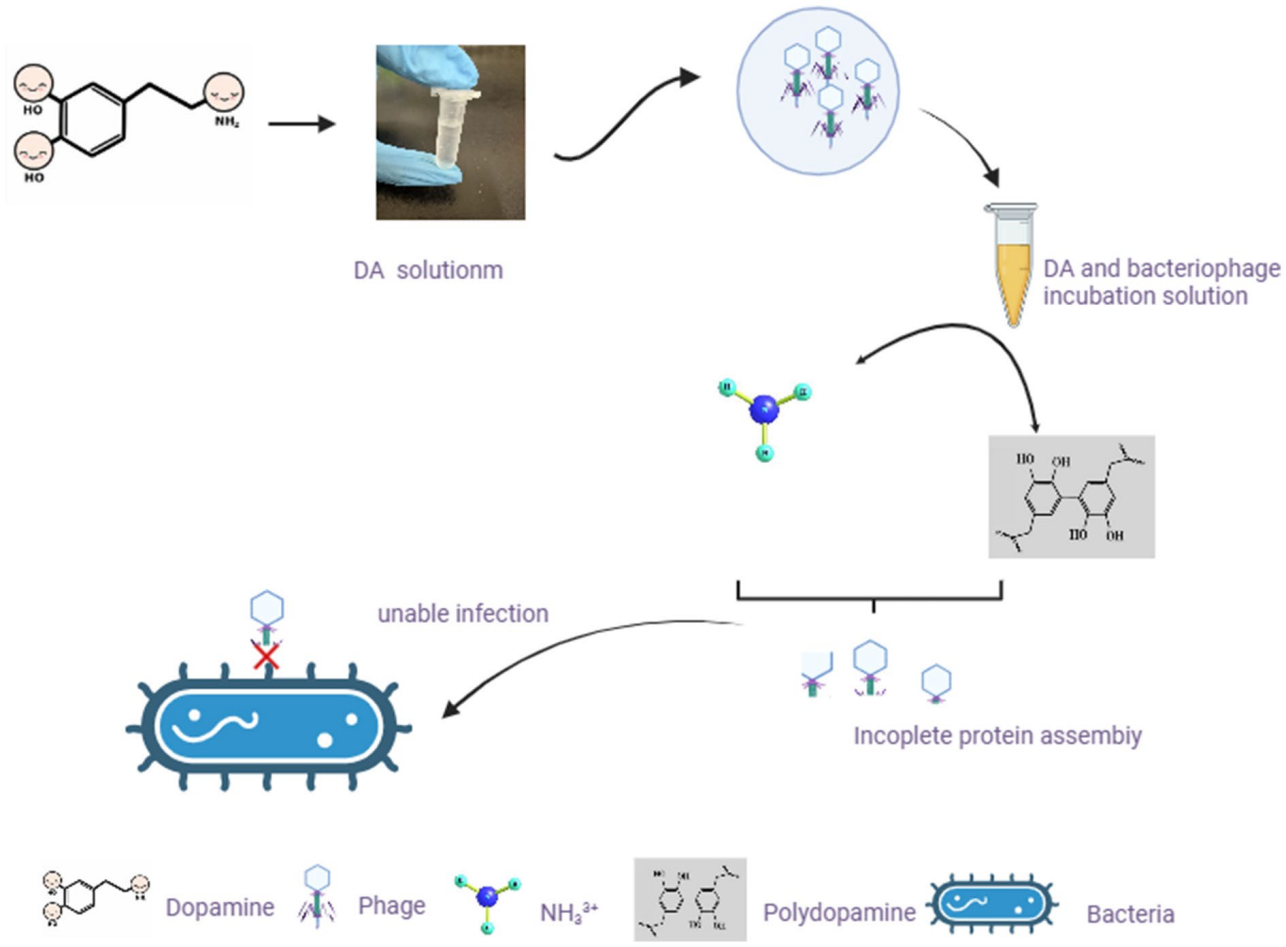
Hypothetical diagram of DA changing the morphology of bacteriophages and exerting their anti-infection mechanisms. In our study, after incubating bacteriophages with DA solution, the morphology of bacteriophages changes. Based on existing knowledge, we predicted two possible scenarios to affect the morphology of bacteriophages, resulting in the inability of bacteriophages to adsorb on the bacterial surface.

DA mainly affects the adsorption of bacteriophages to host bacteria by disrupting their structure. From a chemical perspective, the pH value of the DA solution is approximately 7.0–8.5. In this case, -NH_2_ on DA molecules mainly exists in the form of NH_3_^3+^, and the presence of a certain ion strength may cause the protein shell of bacteriophages to deform (Wang et al., 2022), thereby affecting adsorption. Another possibility is that a certain concentration of DA monomers self-polymerize to obtain polydopamine, which exhibits strong adhesion and forms a strong interaction force with the protein shell of the bacteriophage. This force is between covalent and non-covalent bonds, affecting the assembly of the bacteriophage protein shell and making it difficult to adsorb onto the bacterial surface (Lin et al., 2007). In Figure 8, we predicted the mechanism by which DA affects phage infection based on existing knowledge and results. As DA can facilitate resistance to bacteriophage infection in the host, further studies are required to investigate the application of DA to treat inflammation caused by disorders of the intestinal microbial system.

Recently, research on dopamine has not been limited to neurotransmitters, and several studies have focused on its role in microorganisms. Microorganisms produce, modify, and respond to the same neurochemicals utilized in various signaling pathways in their mammalian hosts. This is the mechanism by which the host and microbiota interact to influence the progression of infectious diseases and behavior through the microbiota-gut-brain axis (Samson et al., 2013; Van Houte et al., 2016). Bacteriophages exist in the presence of bacteria; however, the effects of catecholamines on bacteriophages have not been reported. The relationship between catecholamines and bacteriophages is very complex, with much remaining unknown, and the role of DA in the defense and anti-defense of bacteriophage systems similarly remains unclear, which warrants further exploration.

## Data availability statement

The original contributions presented in the study are included in the article/supplementary material, further inquiries can be directed to the corresponding author.

## Author contributions

XH: Conceptualization, Data curation, Methodology, Supervision, Writing – original draft, Formal analysis. CZ: Formal analysis, Methodology, Supervision, Writing – original draft. YJ: Methodology, Supervision, Formal Analysis, Project administration, Writing – review & editing. XL: Conceptualization, Investigation, Writing – review & editing. SZ: Conceptualization, Investigation, Data curation, Methodology, Supervision, Writing – original draft. YW: Conceptualization, Funding acquisition, Project administration, Resources, Validation, Visualization, Writing – original draft, Data curation.
